# Perceptions of Wild Pig Impact, Management, and Policy in Alabama

**DOI:** 10.1007/s00267-024-01956-8

**Published:** 2024-03-11

**Authors:** Ellary TuckerWilliams, Christopher A. Lepczyk, Wayde Morse, Mark Smith

**Affiliations:** 1 College of Forestry, Wildlife and Environment, Auburn University, Auburn, AL 36849 USA; 2 Present Address: Idaho Department of Fish and Game, Boise, ID USA

**Keywords:** Feral pig, Human dimensions, Wild hog, Wild boar, Land use

## Abstract

Successful management of invasive species often requires working across public and private landownerships. A prime example of an invasive species that commonly occurs on privately and publicly owned and managed lands is the wild pig (*Sus scrofa*). Because of the multitude of negative impacts associated with wild pigs, management must occur across both private and public lands to achieve widespread control and sustained success. However, managing wild pigs across property boundaries is challenging as we know very little about differing management practices and landowner perspectives. To address this knowledge gap, we sought to understand wild pig management efforts on privately owned lands, the perceived economic, ecological, and human health impact of wild pigs, and beliefs related to policy. Generally, stakeholders believe wild pigs have negative impacts on wildlife, the economy, and ecological and public health, however less than half of landowners participate in wild pig control. Furthermore, stakeholders believe that the responsibility of managing and paying for damages associated with wild pigs lies with individual landowners. Our findings suggest that increased efficacy of wild pig control and collaboration between private and public landowners is not only possible but also necessary if wild pig population control is to be regionally successful.

## Introduction

Invasive species are responsible for decreases in global biodiversity (Wilcove et al. 1998; Simberloff et al. 2005; Pysek and Richardson 2010), economic loss (Pimentel et al. 2005; Simberloff et al. 2005; Pejchar and Mooney 2009), ecological degradation (Mack et al. 2000; Pejchar and Mooney 2009; Pysek and Richardson 2010; Barrios-Garcia and Ballari 2012), diminishing ecosystem services (Mack et al. 2000; Pimentel et al. 2005; Pejchar and Mooney 2009; Pysek and Richardson 2010), and deteriorating human health (Mack et al. 2000; Pejchar and Mooney 2009; Pysek and Richardson 2010; Barrios-Garcia and Ballari 2012). Given these negative impacts, significant effort has been expended on managing invasive species. In general, philosophies surrounding invasive species management take one of three approaches, top-down (e.g., command and control), bottom-up (e.g., grassroots), or middle-out (e.g., civic environmentalism) management, specifically tailored to the species of concern (John et al. 2006; Epanchin-Niell et al. 2010). Individually, each of these approaches has its own set of strengths and weaknesses, but collectively, each philosophy and associated management methods uniquely contributes at different levels of management for more effective regional control of biological invasions (John et al. 2006; Epanchin-Niell et al. 2010).

One invasive species of high global concern is the wild pig (*Sus scrofa*, Lowe et al 2000). In the US, wild pigs are one of the most economically costly (Pimentel 2007; Anderson et al. 2016; Holderieath 2016; Anderson et al. 2019; McKee et al. 2020, Fantle-Lepczyk et al. 2022) and ecologically destructive (Siemann et al. 2009; Jolley et al. 2010; Barrios-Garcia and Ballari 2012; Bevins et al. 2014) invasive species. Much of the wild pig population in the continental US is concentrated in the Southeast (McClure et al. 2015), particularly on privately owned land. As a result, private landowners directly experience many of the negative environmental, economic, human health, and wildlife impacts associated with wild pigs. Furthermore, the burden of controlling the wild pig falls heavily on private landowners, as agency personnel and other wild pig removal services are unable to access private property for population control without consent.

Previous research on the human dimensions of wild pigs has focused on quantifying their economic impact. Specifically, multiple studies have each concluded that wild pigs are expensive to landowners and the economy (Adams et al. 2005; Pimentel et al. 2005; Higginbotham et al. 2006; Mengak 2012; Anderson et al. 2016; Poudyal et al. 2017; McKee et al. 2020; Leivers et al. 2023). One Tennessee study found that wild pigs cost landowners collectively $28.31 million in damages and control costs (Poudyal et al. 2017), while a separate study in Texas found that the average landowner experienced an economic loss of approximately $10,146 in damages and management effort (Adams et al. 2005). Likewise, a sample of 8707 licensed hunters who were also landowners in Texas reported average losses of $1696 due to wild pigs while generating only $23.36 of income from wild pigs (Leivers et al. 2023). Furthermore, Mengak (2012) found that the average respondent lost $12,646 in crop associated damages and loss, in addition to an average loss of $5381 to non-crop related items. Contrastingly, a small number of individuals do benefit from monies earned via wild pig hunting opportunities (Rollins 1993; Tolleson et al. 1995; Adams et al. 2005). However, Leivers et al. (2023) concluded the economic benefits of wild pig control (i.e., damage reduction) in Texas were far greater than income generated through provision of hunting opportunities by landowners.

Beyond the economic evaluations, prior research has sought to understand stakeholders’ perspectives on the wild pig impacts on the environment, human health, and other wildlife species (Rollins 1993; Adams et al. 2005; Mengak 2012; Harper et al. 2014; Harper et al. 2016; Caplenor et al. 2017). Specific to environmental impacts, previous research asked broadly if respondents believed wild pigs had an impact on the environment, of which the majority agreed they were environmentally harmful. Respondents were not asked to identify specific details on the exact type of effect (Adams et al. 2005; Mengak 2012; Harper et al. 2014; Harper et al. 2016; Caplenor et al. 2017).

Regarding the impact wild pigs have on human health, few studies asked stakeholders if they believed wild pigs were a source of disease and found varying levels of agreement. Studies in Georgia and Illinois found that 61% (Mengak 2012) and 73% (Harper et al. 2016) of respondents believed wild pigs were a source of disease, respectively. Contrastingly, a study in Texas found that only 34% of respondents believed wild pigs to be a disease hazard (Adams et al. 2005). Additionally, Mengak (2012) and Harper et al. (2016) asked respondents if they believed wild pigs threatened public safety, of which 67% and 77% agreed, respectively.

Conflicting results and a small number of studies suggest more research is necessary. In relation to the impact wild pigs have on other species, previous studies had predominately addressed preferred game species (e.g., white-tailed deer, turkey, and bobwhite quail) and found a perceived negative association with wild pigs (Rollins 1993; Adams et al. 2005; Mengak 2012; Harper et al. 2014). In Texas, McLean et al. (2021) reported that 83% of hunters had a low tolerance for wild pigs with most hunters favoring substantial population reductions. Except for Mengak (2012), these studies overlooked wild pig impact on non-game species. Mengak (2012) found that less than 20% of respondents believed wild pigs negatively impacted the gopher tortoise (*Gopherus polyphemus*), and less than 10% believed wild pigs negatively affected waterfowl (*Anseriformes* spp.) and songbirds (*Passeri* spp.). Additionally, previous studies have broadly assessed wild pig management on private property (Adams et al. 2005; Higginbotham et al. 2006; Mengak 2012; Harper et al. 2014; Anderson et al. 2016; Caplenor et al. 2017) and found that stakeholders were accepting of and largely engage in lethal removal techniques, such as hunting and trapping (Adams et al. 2005; Higginbotham et al. 2006; Mengak 2012; Harper et al. 2014; Anderson et al. 2016; Caplenor et al. 2017). However, stakeholders view current methods of wild pig population control to be ineffective (Mengak 2012).

Despite multiple studies assessing the human dimensions of wild pig damage, impact, and management, large gaps still exist within the literature. Much of the previous wild pig human dimensions work has been broad in scope and lacks specificity. By gaining a more thorough understanding of stakeholders’ perspectives on wild pig populations and trends on private lands, efficacy of general wild pig management, perceived impacts of wild pigs, and attitudes towards current policy, managers can gain a more holistic view of the issues surrounding wild pigs and wild pig population control. With such information, managers would be able to improve overall wild pig management effort and effectiveness.

To address this knowledge gap, our goal was to better understand stakeholder’s perspectives on wild pig impact on private land, general wild pig management, and wild pig related policy. Specifically, our objectives were to: 1) determine stakeholder perceptions on wild pig populations and trends across landownerships; 2) gain a detailed understanding of private land management of wild pigs; 3) determine stakeholder beliefs regarding the impact wild pigs have on the environment, economy, human health, and wildlife; 4) quantify stakeholders’ perspectives towards current wild pig related policy; and, 5) determine if perspectives differed between stakeholder group (hunter, farmer, and forestland owner). We addressed these objectives across the state of Alabama as it has been markedly affected by wild pigs (McKee et al. 2020; TuckerWilliams et al. 2021) and represents an ideal location to evaluate stakeholder views and management options.

## Methods

To address our objectives, we created a social survey instrument consisting of 58 questions, 14 of which pertained to wild pig population trends, management, impact, or policy (Appendix 1). Survey questions regarding respondents’ perceptions of wild pig population trends addressed questions such as perceived current population level of wild pigs on respondents’ property, population trends over time, and perceived reasons for observed population trends. Additionally, survey questions pertaining to wild pig management on private property addressed the types of management methods that were being utilized, and perceived effectiveness of such methods. Questions relating to the impact of wild pigs addressed stakeholder beliefs on the economic, ecological, and human health impact of wild pigs in addition to the impact on other wildlife species. Finally, survey questions relating to wild pig policy addressed respondents’ beliefs on who should ultimately be responsible for managing wild pigs and paying for associated damages, and satisfaction with current legal repercussions for the transportation of live wild pigs (Appendix 1). Previous surveys on stakeholder perspectives towards wild pigs provided a basis for survey questions and design (Mengak 2012, Harper et al. 2014; Mississippi State University, Wild Hog Public Attitude Survey, unpublished).

The survey instrument was designed to be disseminated to three key stakeholder groups, hunters, farmers, and forestland owners throughout Alabama. These three groups were selected because they own most of the private land within Alabama (US Census Bureau 1991; Hartsell and Johnson 2005; USDA NASS 2018) and therefore are the most likely to interact with and be affected by wild pigs in the state. The draft survey was peer-reviewed in a pilot study of 10 volunteers from the School of Forestry and Wildlife Sciences at Auburn University, and reviewed by the Alabama Farmers Federation (ALFA) and Alabama Forest Owners Association (AFOA) to improve the quality of the survey instrument which was subsequently revised. The final survey was approved by the Auburn University Institutional Review Board (IRB) (Protocol #17-397 EX 1710), prior to administration.

Following the Tailored Design Method (Dillman et al. 2009), we administered the survey via the Internet in January 2018, using Qualtrics. An invitation email with the link to the survey was disseminated to each of the three stakeholder groups using email addresses of the group members followed by two reminder emails at two and four weeks after the initial email. Specifically, emails were sent by ALFA to all Alabama row crop, produce, hay, cattle, domestic pig, poultry, and sheep farmers within the ALFA membership list which equated to approximately 10,700 individual farmers. To survey hunters, we purchased 5000 email addresses of individuals who had purchased an Alabama hunting license for the 2017–2018 season from the Alabama Department of Conservation and Natural Resources (ADCNR). However, only 4621 of the 5000 email addresses were valid due to duplicates and obsolete email addresses. Finally, the AFOA distributed the email to all members who owned forestland in Alabama, approximately 4000 individuals. In total approximately 19,321 people received invitations to participate in the survey. To differentiate between stakeholders each group received a separate and unique online link to the survey.

We did not test for non-response bias due to format of the invitation and data collection requirements of both the software and human subjects’ requirements. Specifically, IP addresses of respondents were not collected in order to protect respondent anonymity in accordance with the Auburn University IRB. Therefore, we were unable to use that information for non-response bias testing. Additionally, because ALFA and AFOA did not want to release the contact information of their members, each organization sent the survey in an email directly to their members.

All survey respondents were required to provide consent in order to gain access to the survey, thereby acknowledging that they had read the consent letter, verifying that they were at least 19 years of age and agreeing to participate in the research project. At the end of the survey individuals were given the opportunity to provide their email address if they wanted to receive a summary of the survey results. To increase response rates, the survey was incentivized. At the completion of the survey, individuals were given the option to submit their name and mailing address in a prize drawing to win 1 of 5 Amazon gift cards, each valued at $100. The survey was closed at the beginning of March 2018.

The format of the survey included binary, fill in the blank, multiple choice, select all that apply, and five-point Likert scale questions. Variation between question formatting occurred in accordance with the specifics of the question being asked. “Unsure” responses were excluded from analyses and treated as missing data. We reviewed “Other” responses that survey participants provided, of which nearly all were already encompassed by the participant’s previous answers in the corresponding question. A small percentage of answers that were not encompassed by the options provided in the survey question were excluded from analysis and treated as missing. When survey respondents were asked to identify how much they agreed with the statements: “I believe that wild pigs…. a) have a positive ecological impact, b) improve soil quality by rooting, and c) improve wildlife habitat” the scale was reversed in order to achieve uniform directionality within the question and reduce confusion when discussing the results.

Initial statistical analysis consisted of descriptive statistics of all questions. An important note, not all respondents were required to answer all questions in the survey, therefore response rate varied by question. Since one of the objectives of the survey was to determine whether perspectives towards wild pigs, wild pig management, and policy differed among hunters, forestland owners, and farmers, a one-way ANOVA was used to test for significant differences among the means of those stakeholder groups. Any significant differences determined by the one-way ANOVA were then tested using the Turkey’s post-hoc comparison. A total of 14 questions were analyzed, of which an ANOVA was used on 10 questions to determine significant differences among stakeholder groups. The four remaining questions were analyzed with descriptive statistics including count data and percentages. ANOVA results are presented as means ± standard deviation. All statistical analyses were conducted in accordance with Vaske (2008) and run in SPSS 24 (IBM Corp. Released 2016) with *p*-value ≤ 0.05 considered significant.

The overall response rate was approximately 9% (n = 1822) and varied by stakeholder group. A total of 668 hunters, 1055 farmers, and 99 forestland owners responded, equating to a 14, 10, and 2% response rate, respectively. The low response rate of forestland owners is likely due to a difference in survey methodology by AFOA. Specifically, AFOA did not send a specific email inviting members to participate in the survey over concerns of spamming and instead included the survey invitation as part of a general email that also contained additional information associated with the AFOA. Therefore, AFOA members likely did not notice the survey option within the body of the email. However, despite the low sample size, forestland owner responses were similar to the other two groups and therefore are included in the analyses, but contextualized based on their sample size (TuckerWilliams et al. 2021).

## Results

### Demographics

The average respondent was in their late 50’s, Caucasian, male, had a household income between $75,000 and $99,999 in 2017, and had lived in Alabama for roughly 53 years. Most respondents had some form of higher education and most owned land in Alabama. Respondents lived in urban, suburban, and rural communities, with ~29% living in a town or city with many neighbors, ~28% living in an area outside of a town with scattered neighbors, and~43% living in a rural area with few neighbors (n = 1705). Respondents were from every county in Alabama, with Baldwin, Mobile, Jefferson, and Tuscaloosa County having the greatest number of respondents. (See TuckerWilliams et al. 2021 for full description of stakeholders’ demographics).

### Population Trend on Private Lands

**Fig. 1:**
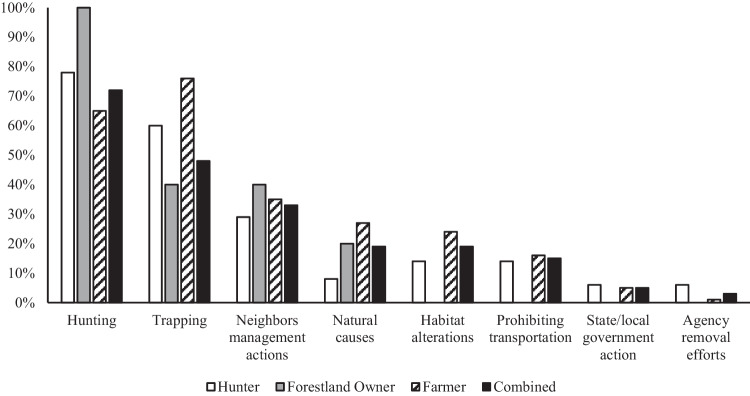
Property owners perceived reasons for decreasing wild pig populations on their property by stakeholder group. Sample size for each group is as follows, hunter n = 51, forestland owner n = 5, farmers n = 75, combined n = 131

**Fig. 2:**
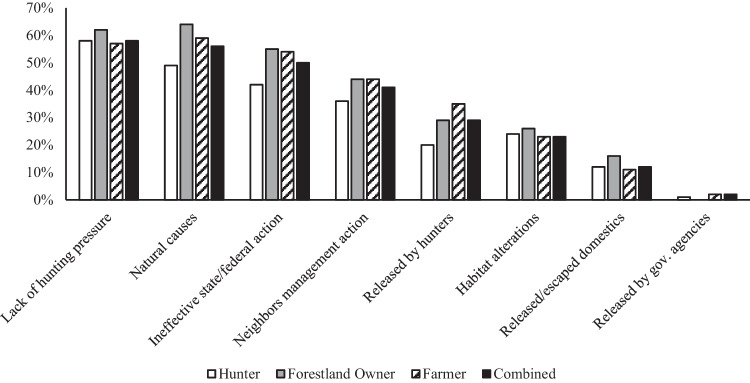
Property owners perceived reasons for increasing wild pig populations on their property by stakeholder group. Sample size for each group is as follows, hunter n = 277, forestland owner n = 55, farmer n = 415, combined n = 747

On average hunters, forestland owners, and farmers indicated that they sometimes saw wild pigs on their property(ies), and therefore, believed they had approximately medium wild pig population levels across their properties (1.8 ± 0.8; Table 1). However, the mean Likert scale score for wild pig population level on properties owned by forestland owners (2.2 ± 0.8) was significantly greater than both farmers and hunters (Table 1). When participants were asked if they believed the wild pig populations across all their land had increased, decreased, or stayed the same over the past 5 years, forestland owners indicated that they had noticed a significantly greater increase in wild pigs on their property(ies) (4.0 ± 1.1) than both hunters (3.6 ± 1.1) and farmers (3.6 ± 1.0, Table 1). Individuals who stated that they believed wild pig populations had generally decreased on their property(ies) over the past five years were then asked to identify the reasons for this observed population trend. Hunting (~71%), trapping (~48%), and the neighboring property’s management actions (33%) were identified as top reasons for decreased wild pig populations (n = 131; Fig. 1). Comparatively, individuals who stated that they had observed an increase in wild pig population on their property(ies) over the past five years were also asked to identify the perceived reasons why. Lack of hunting pressure (~58%), natural causes such as increased food or water availability or natural dispersal (~56%), and ineffective action taken by state and/or federal agencies to remove wild pigs (~50%) were selected as the principal reasons for increased wild pig populations (n = 747; Fig. 2).

**Table 1:** Mean Likert scores with ANOVA comparisons and Turkey’s post hoc tests by stakeholder group

Question		Grand Mean ± SD (n)	F(df)	*p*-value	Hunter	Farmer	Forestland Owner
Stakeholders perspective on wild pig population trends across their property(ies)	Current wild pig population level across all properties^a^	1.8 ± 0.8 (n = 1420)	11.50 (2,1417)	0.000^j,k^	1.8 ± 0.8 (n = 520)	1.8 ± 0.8 (n = 817)	2.2 ± 0.8 (n = 85)
	Wild pig population trend over the past 5 years^b^	3.6 ± 1.0 (n = 1502)	5.43 (2,1499)	0.004^j,k^	3.6 ± 1.1 (n = 554)	3.6 ± 1.0 (n = 863)	4.0 ± 1.1 (n = 83)
Stakeholders choice to partake in wild pig management^c^ and the perceived effectiveness of current legal wild pig management options^d^ aimed at reducing populations	Engaged in wild pig management within the past 5 years	1.6 ± 0.5 (n = 1599)	13.38 (2,1596)	0.00^j,k^	1.6 ± 0.5 (n = 591)	1.6 ± 0.5 (n = 919)	1.3 ± 0.5 (n = 89)
	Nuisance permit for hunting	3.4 ± 1.3 (n = 1481)	14.89 (2,1478)	0.000^j,k,l^	3.6 ± 1.2 (n = 543)	3.3 ± 1.3 (n = 857)	2.8 ± 1.3 (n = 81)
	Night shooting	3.8 ± 1.1 (n = 1484)	19.12 (2,1481)	0.000^j,l^	4.0 ± 1.1 (n = 543)	3.7 ± 1.1 (n = 860)	3.4 ± 1.3 (n = 81)
	Trapping and lethal removal (e.g., corral traps)	4.1 ± 1.0 (n = 1481)	4.42 (2,1478)	0.012^l^	4.2 ± 0.9 (n = 541)	4.0 ± 1.0 (n = 859)	3.9 ± 1.0 (n = 81)
	Opportunistic shooting (not actively seeking out wild pigs but shooting them if the opportunity presents itself)	3.3 ± 1.4 (n = 1483)	11.84 (2,1480)	0.000^j,l^	3.5 ± 1.3 (n = 544)	3.2 ± 1.3 (n = 859)	2.9 ± 1.5 (n = 80)
	Management cooperatives (groups of landowners who come together and share in the costs and labor of removing wild pigs from their collective land)	3.6 ± 1.0 (n = 1479)	2.73 (2,1476)	0.066	3.6 ± 1.1 (n = 542)	3.6 ± 1.0 (n = 857)	3.3 ± 1.1 (n = 80)
	For hire private removal service	3.4 ± 1.0 (n = 1475)	0.99 (2,1472)	0.373	3.4 ± 1.1 (n = 539)	3.4 ± 1.0 (n = 855)	3.3 ± 1.1 (n = 81)
	Hunting/shooting over bait	4.1 ± 1.1 (n = 1480)	25.24 (2,1477)	0.000^j,l^	4.3 ± 1.0 (n = 543)	3.9 ± 1.1 (n = 857)	3.7 ± 1.3 (n = 80)
	Aerial shooting by helicopter	3.5 ± 1.2 (n = 1471)	11.42 (2,1468)	0.00 0^j,l^	3.7 ± 1.2 (n = 538)	3.4 ± 1.2 (n = 853)	3.3 ± 1.2 (n = 80)
	Fencing (including electric)	3.0 ± 1.2 (n = 1467)	2.90 (2,1464)	0.056	3.1 ± 1.2 (n = 536)	3.0 ± 1.2 (n = 851)	2.9 ± 1.3 (n = 80)
	Scare tactics (e.g., motion activated scarecrows, scents to deter wild pigs)	2.3 ± 1.1 (n = 1472)	0.84 (2,1469)	0.432	2.3 ± 1.2 (n = 539)	2.3 ± 1.1 (n = 852)	2.2 ± 1.1 (n = 81)
	Habitat alteration (e.g., burning to remove understory)	2.9 ± 1.1 (n = 1470)	0.16 (2,1467)	0.855	2.9 ± 1.1 (n = 537)	2.9 ± 1.1 (n = 853)	2.9 ± 1.1 (n = 80)
Belief in who should be responsible for managing wild pig populations throughout Alabama^e^	The extension service	3.1 ± 1.3 (n = 1509)	1.05 (2,1506)	0.349	3.1 ± 1.3 (n = 549)	3.2 ± 1.3 (n = 875)	3.0 ± 1.2 (n = 85)
	Federal agencies (e.g., USDA, Fish and Wildlife Service)	3.0 ± 1.4 (n = 1509)	7.11 (2,1506)	0.001^k,l^	2.9 ± 1.4 (n = 549)	3.1 ± 1.4 (n = 874)	2.6 ± 1.5 (n = 86)
	Hunters	4.0 ± 1.1 (n = 1507)	57.41 (2,1504)	0.000^j,l^	4.4 ± 0.9 (n = 556)	3.8 ± 1.2 (n = 865)	3.8 ± 1.1 (n = 86)
	Individual landowners	4.5 ± 0.8 (n = 1523)	2.69 (2,1520)	0.068	4.5 ± 0.8 (n = 556)	4.4 ± 0.9 (n = 881)	4.4 ± 1.0 (n = 86)
	The private industry (e.g., for hire wild pig removal companies)	3.1 ± 1.3 (n = 1503)	0.98 (2,1500)	0.375	3.1 ± 1.3 (n = 547)	3.1 ± 1.3 (n = 870)	2.9 ± 1.4 (n = 86)
	State agencies (e.g., Alabama Department of Conservation and Natural Resources)	3.6 ± 1.2 (n = 1516)	1.68 (2,1513)	0.187	3.6 ± 1.3 (n = 554)	3.6 ± 1.3 (n = 85)	3.6 ± 1.3 (n = 85)
	The general public	2.9 ± 1.4 (n = 1501)	26.85 (2,1548)	0.000^j,l^	3.2 ± 1.4 (n = 548)	2.7 ± 1.5 (n = 84)	2.7 ± 1.5 (n = 84)
Stakeholders perceived level of importance that authorities address the concerns of the following groups of people regarding wild pig management in Alabama^f^	Animal welfare groups (e.g., The Humane Society, PETA)	2.1 ± 1.4 (n = 1493)	6.58 (2,1490)	0.001^l^	1.9 ± 1.3 (n = 546)	2.2 ± 1.4 (n = 867)	2.0 ± 1.3 (n = 80)
	Biologists, wildlife/land managers, scientists	4.1 ± 1.0 (n = 1495)	4.25 (2,1492)	0.014^l^	4.2 ± 0.9 (n = 548)	4.0 ± 1.1 (n = 865)	4.1 ± 1.0 (n = 82)
	Farmers/agricultural professionals	4.6 ± 0.8 (n = 1491)	1.19 (2,1488)	0.151	4.5 ± 0.8 (n = 544)	4.6 ± 0.8 (n = 865)	4.7 ± 0.7 (n = 82)
	Forestland owners	4.6 ± 0.8 (n = 1490)	2.55 (2,1487)	0.079	4.5 ± 0.8 (n = 544)	4.6 ± 0.8 (n = 864)	4.7 ± 0.7 (n = 82)
	Hunters	4.0 ± 1.1 (n = 1491)	50.72 (2,1488)	0.000^j,l^	4.4 ± 0.9 (n = 548)	3.8 ± 1.2 (n = 861)	3.9 ± 1.2 (n = 82)
	Landowners	4.6 ± 0.8 (n = 1490)	0.65 (2,1487)	0.523	4.6 ± 0.7 (n = 545)	4.6 ± 0.8 (n = 863)	4.7 ± 0.8 (n = 82)
	The general public	3.2 ± 1.2 (n = 1478)	2.55 (2,1475)	0.078	3.2 ± 1.2 (n = 540)	3.1 ± 1.3 (n = 857)	3.1 ± 1.2 (n = 81)
	Public land recreational users (e.g., hikers, birders, horseback riders)	3.4 ± 1.2 (n = 1484)	0.83 (2,1481)	0.437	3.5 ± 1.2 (n = 541)	3.4 ± 1.2 (n = 862)	3.5 ± 1.3 (n = 81)
	Wild pig related businesses (e.g., guided pig hunts, removal companies, wild game meat processors)	3.1 ± 1.4 (n = 1491)	12.27 (2,1488)	0.000^j,l^	3.3 ± 1.3 (n = 543)	3.0 ± 1.3 (n = 866)	2.7 ± 1.4 (n = 82)
Stakeholders belief on whether or not the penalties for individuals caught transporting or releasing live wild pigs in Alabama are sufficient or insufficient^g^	Class B misdemeanor	1.6 ± 0.6 (n = 1474)	6.98 (2,1471)	0.001^j,l^	1.7 ± 0.6 (n = 535)	1.6 ± 0.6 (n = 859)	1.5 ± 0.6 (n = 80)
	Mandatory fine of $2500 per wild pig	1.7 ± 0.6 (n = 1481)	4.87 (2,1478)	0.008^j,l^	1.8 ± 0.6 (n = 538)	1.7 ± 0.6 (n = 861)	1.6 ± 0.6 (n = 82)
	Up to 180 days in jail	1.9 ± 0.7 (n = 1463)	1.00 (2,1460)	0.369	2.0 ± 0.7 (n = 526)	1.9 ± 0.7 (n = 856)	1.9 ± 0.7 (n = 81)
Stakeholders beliefs on why wild pigs are an economic issue in Alabama^h^	Cost individuals money	3.8 ± 1.2 (n = 1680)	18.46 (2,1677)	0.000^j,k,l^	3.6 ± 1.2 (n = 621)	3.9 ± 1.0 (n = 967)	4.2 ± 1.0 (n = 92)
	Kill newborn livestock	3.5 ± 1.1 (n = 1675)	2.97 (2,1672)	0.051^l^	3.4 ± 1.1 (n = 620)	3.6 ± 1.0 (n = 965)	3.6 ± 0.9 (n = 90)
	Economically costly to the state	4.3 ± 1.1 (n = 1672)	16.15 (2,1669)	0.000^j,l^	4.2 ± 1.2 (n = 614)	4.4 ± 1.1 (n = 965)	4.6 ± 0.8 (n = 93)
Stakeholders beliefs on why wild pigs are a human health issue in Alabama^h^	Threaten human health	4.0 ± 1.1 (n = 1675)	33.50 (2,1672)	0.000^j,l^	3.7 ± 1.2 (n = 618)	4.1 ± 1.0 (n = 967)	4.3 ± 0.8 (n = 90)
	Transmit disease to humans	3.5 ± 1.0 (n = 1673)	12.06 (2,1670)	0.000^j,l^	3.3 ± 1.0 (n = 620)	3.6 ± 1.0 (n = 964)	3.7 ± 0.9 (n = 89)
	Threaten public safety	3.9 ± 1.1 (n = 1670)	29.61 (2,1667)	0.000^j,l^	3.6 ± 1.2 (n = 615)	4.0 ± 1.1 (n = 964)	4.0 ± 0.9 (n = 91)
	Transmit disease to domestic livestock	3.8 ± 1.0 (n = 1677)	22.46 (2,1664)	0.000^l^	3.6 ± 1.0 (n = 620)	3.9 ± 1.0 (n = 966)	3.8 ± 0.9 (n = 91)
Stakeholders beliefs on why wild pigs are an ecological issue in Alabama^h^	Negative ecological impact	4.0 ± 1.4 (n = 1667)	10.40 (2,1665)	0.000^j,k^	3.9 ± 1.4 (n = 615)	4.1 ± 1.4 (n = 961)	4.6 ± 1.0 (n = 91)
	Reduce quality of water sources	4.1 ± 1.1 (n = 1674)	13.29 (2,1671)	0.000^j,l^	3.9 ± 1.2 (n = 616)	4.2 ± 1.1 (n = 968)	4.4 ± 0.9 (n = 90)
	Decreased soil quality by rooting	4.2 ± 1.2 (n = 1667)	2.80 (2,1665)	0.061	4.1 ± 1.2 (n = 612)	4.2 ± 1.2 (n = 964)	4.3 ± 1.1 (n = 91)
	Cause tree loss and damage	4.0 ± 1.2 (n = 1670)	8.17 (2,1668)	0.000^j,l^	3.9 ± 1.2 (n = 612)	4.1 ± 1.1 (n = 966)	4.3 ± 1.1 (n = 92)
	Decreased wildlife habitat	4.4 ± 1.1 (n = 1667)	4.48 (2,1665)	0.011^j,k^	4.4 ± 1.1 (n = 612)	4.4 ± 1.1 (n = 963)	4.7 ± 0.8 (n = 92)
Stakeholders beliefs on whether or not wild pigs have a positive or negative impact on native wildlife in Alabama^i^	Amphibians	2.1 ± 1.0 (n = 1266)	12.00 (2,1263)	0.000^j,l^	2.3 ± 1.1 (n = 477)	2.0 ± 1.0 (n = 706)	1.9 ± 0.9 (n = 83)
	Endangered species	2.2 ± 1.0 (n = 1220)	13.08 (2,1217)	0.000^j,l^	2.3 ± 1.0 (n = 451)	2.1 ± 1.0 (n = 696)	2.0 ± 1.0 (n = 73)
	Fish	2.4 ± 0.9 (n = 1244)	7.60 (2,1241)	0.001^l^	2.5 ± 0.8 (n = 461)	2.3 ± 0.9 (n = 706)	2.3 ± 0.8 (n = 77)
	Game birds	1.9 ± 1.1 (n = 1409)	11.31 (2,1406)	0.000^j,l^	2.1 ± 1.1 (n = 520)	1.8 ± 1.1 (n = 802)	1.6 ± 0.9 (n = 87)
	Predators/carnivores	2.7 ± 0.9 (n = 1281)	2.00 (2,1278)	0.141	2.7 ± 0.9 (n = 472)	2.6 ± 0.9 (n = 731)	2.6 ± 0.8 (n = 78)
	Reptiles	2.2 ± 1.1 (n = 1346)	11.91 (2,1343)	0.000^j,k,l^	2.4 ± 1.1 (n = 507)	2.2 ± 1.1 (n = 755)	1.9 ± 0.8 (n = 84)
	Small mammals	2.2 ± 0.9 (n = 1361)	9.77 (2,1358)	0.000^j,l^	2.3 ± 1.0 (n = 500)	2.1 ± 0.9 (n = 779)	2.0 ± 0.9 (n = 82)
	Waterfowl	2.3 ± 0.9 (n = 1316)	3.20 (2,1313)	0.041^l^	2.4 ± 0.9 (n = 487)	2.3 ± 0.9 (n = 752)	2.2 ± 0.9 (n = 77)
	White-tailed deer	1.8 ± 1.0 (n = 1463)	2.28 (2,1461)	0.10	1.9 ± 1.2 (n = 5 53)	1.8 ± 1.0 (n = 823)	1.6 ± 0.9 (n = 87)
	Wild Turkey	1.7 ± 1.1 (n = 1486)	7.04 (2,1483)	0.00^j,l^	1.8 ± 1.2 (n = 559)	1.6 ± 1.1 (n = 838)	1.4 ± 0.8 (n = 89)

^a^Multiple choice, 1 = low (I rarely see wild pigs on my property), 2 = medium (I sometimes see wild pigs on my property), 3 = high (I see wild pigs on my property frequently)

^b^5 point Likert scale (1 = largely decreased, 3 = stayed the same, 5 = largely increased)

^c^Polar scale (1 = yes, 2 = no)

^d^5 point Likert scale (1 = very ineffective, 3 = neutral, 5 = very effective)

^e^5 point Likert scale (1 = strongly disagree, 3 = neutral, 5 = strongly agree)

^f^5 point Likert scale, (1 = extremely unimportant, 3 = neutral, 5 = extremely important)

^g^Multiple choice (1 = no, the penalty needs to be stronger, 2 = yes, this is a sufficient penalty, 3 = no, the penalty is too strong)

^h^5 point Likert scale (1 = strongly disagree, 3 = neutral, 5 = strongly agree)

^i^5 point Likert scale (1 = strong negative impact, 3 = neutral/no impact, 5 = strong positive impact)

^j^Hunters and forestland owners significantly differ

^k^Forestland owners and farmers significantly differ

^l^Farmers and hunters significantly differ

### Wild Pig Management

**Fig. 3:**
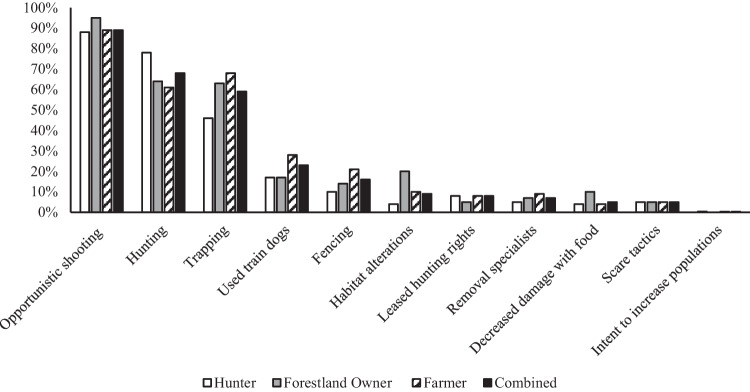
Wild pig management techniques utilized by stakeholders their privately owned or leased property within the last 5 years. Sample size is as follows, hunter n = 239, forestland owner n = 59, farmer n = 352, and combined groups n = 650

When asked if they had engaged in any wild pig management within the last 5 years, ~59% (n = 1599) of all survey participants said “no” with a combined mean Likert scale score of 1.6 ± 0.5 (Table 1). Furthermore, a greater percentage of forestland owners participated in wild pig management than hunters and farmers. The ~ 41% (n = 1599) of survey participants who stated that they had participated in wild pig management over the past 5 years were then asked to identify the methods they had utilized. Opportunistic shooting of wild pigs to control for damage or to control populations was the most commonly selected management strategy by all three stakeholder groups (~89%, n = 650; Fig. 3). Hunting for recreation or subsistence (~68%, n = 650), and trapping (~59%, n = 650) were, respectively, the second and third most selected management strategy (Table 1). A follow up question then asked stakeholders to identify their perceived level of effectiveness for “current legal wild pig management options” at reducing wild pig populations. Trapping and lethal removal (4.1 ± 1.0), hunting/shooting over bait (4.1 ± 1.1), and night shooting (3.8 ± 1.1) received the highest combined mean Likert scale scores, indicating that the above stated management options were generally “somewhat effective” (Table 1). Hunters differed from farmers and forestland owners regarding the overall effectiveness of night shooting and hunting/shooting over bait with significantly greater perceived effectiveness (Table 1). Hunters also differed significantly with greater perceived effectiveness of trapping and lethal removal as a wild pig management technique than farmers (Table 1).

### Wild Pig Impact

Specific to the economic impact of wild pigs, all three stakeholder groups agreed with the statement “wild pigs are an issue because they are economically costly to the state” (4.3 ± 1.1), however hunters differed from forestland owners and farmers with a significantly lower mean Likert scale score in comparison (Table 1). Regarding human health, stakeholders “somewhat agree” that “wild pigs are an issue because they threaten human health (e.g., disease, water contamination)” (4.0 ± 1.1), and again hunters were significantly lower in agreeance than forestland owners and farmers (Table 1). Overall, stakeholders believed wild pigs have negative ecological impacts (Table 1), with decreased wildlife habitat (4.4 ± 1.1) and soil quality (4.2 ± 1.2) receiving the highest overall combined mean Likert scale scores (Table 1). Hunters had significantly lower levels of agreement than farmers and forestland owners regarding wild pigs’ ability to reduce water quality and negatively impact trees. Additionally, forestland owners had significantly greater levels of agreement than hunters and farmers in that wild pigs have a negative ecological impact and reduce the quality of wildlife habitat (Table 1).

Regarding the impact wild pigs have on wildlife, survey participants were asked to identify whether they believed that wild pigs have a positive or negative impact on various wildlife species or groupings of wildlife. In no instance did any stakeholder group identify a species or grouping of species that was positively impacted by wild pigs (Table 1). Wild turkey (*Meleagris gallapavo silvertris*) (1.7 ± 1.1), white-tailed deer (*Odocoileus virginianus*) (1.8 ± 1.0), and game birds (e.g., mourning dove [*Zenaida macroura*], bobwhite quail [*Colinus virginianus*]) (1.9 ± 1.1) received the lowest combined mean Likert scale scores of between “slight negative impact” and “strong negative impact” respectively (Table 1). Interestingly, hunters differed from forestland owners and farmers with significantly greater mean Likert scale scores in the wild turkey and game bird categories (Table 1).

### Wild Pig Regulations and Policy

All stakeholder groups agreed in their belief that individual landowners should be responsible for managing wild pig populations throughout Alabama (4.5 ± 0.8), while hunters (4.0 ± 1.14), and state agencies (3.6 ± 1.2) received the subsequent greatest mean Likert scale score (Table 1). Hunters had significantly greater belief that hunters should be responsible for managing wild pigs than farmers and forestland owners (4.4 ± 0.9; Table 1). Interestingly, federal agencies overall received a neutral opinion on whether they should manage wild pigs in Alabama (3.0 ± 1.4), however hunters (2.9 ± 1.4) and forestland owners (2.6 ± 1.5) were significantly lower in their agreeance than farmers (3.1 ± 1.1; Table 1).

**Fig. 4:**
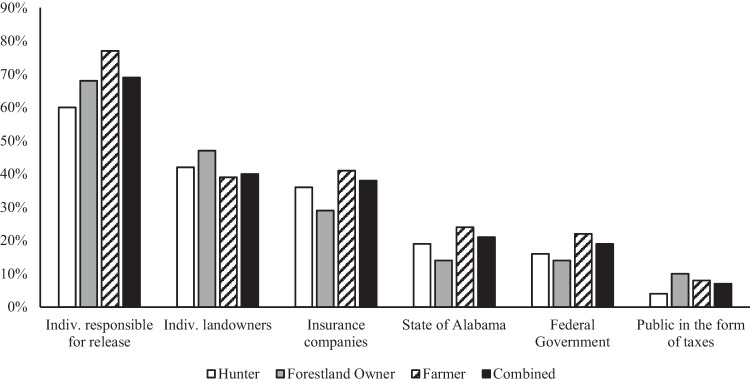
Percentage of respondents indicating who should be responsible for paying for wild pig damages they have experienced on their private property by stakeholder group. Sample size for each group is as follows, hunter n = 549, forestland owner n = 87, farmer n = 836, total n = 1492

Along with the responsibility of wild pig management comes the cost of damage associated with wild pigs, and who is responsible for such costs. Therefore, stakeholders were asked to identify who they believed should be responsible for paying for the damage caused by wild pigs. Of the total number of survey participants who answered this question, ~69% (n = 1492) believed that the individuals responsible for the release of wild pigs should also be responsible for paying for said damages. Interestingly, individual landowners (~40%) and insurance companies (~38%) were the second and third most selected answers while the state of Alabama and the federal government only received ~21 and ~19% of the vote, respectively (n = 1492; Fig. 4).

In addition, stakeholders were asked to identify how sufficient or insufficient the current legal repercussions are for individuals caught transporting or releasing live wild pigs anywhere in Alabama. As it stands, an individual(s) caught transporting live or releasing wild pigs will be charged with a “Class B misdemeanor, mandatory fine of $2500 per wild pig, and up to 180 days in jail.” Farmers, forestland owners and hunters on average believed that a “Class B misdemeanor” (1.6 ± 0.6), and “mandatory fine” (1.7 ± 0.6) to be between “yes, this is a sufficient penalty” and” “no, the penalty needs to be stronger” (Table 1). Hunters differed significantly from forestland owners and farmers on both accounts, believing the penalties to be sufficiently severe (Table 1). All three stakeholder groups agreed that the penalty of “up to 180 days in jail” was sufficient with a combined mean Likert scale score of 1.9 ± 0.7 (Table 1).

Finally, survey participants were asked to identify how important they believed it is for authorities like the Alabama Department of Conservation and Natural Resources to address the concerns of various groups of people regarding wild pig management in Alabama. In descending order, “Land owners” (4.6 ± 0.8), “farmers and agricultural professionals” (4.6 ± 0.8), “forestland owners” (4.6 ± 0.8), “biologist, scientists, wildlife and land managers” (4.1 ± 1.0) and “hunters” (4.0 ± 1.1) each received a combined mean Likert scale score between “somewhat important” to “extremely important” (Table 1). Hunters differed from farmers with significantly greater level of importance for authorities to address the concerns of “biologists, scientists, and wildlife or land managers” regarding wild pig management. Additionally, hunters placed significantly more importance on authorities addressing the concerns of hunters in wild pig management than both farmers and forestland owners (Table 1).

## Discussion

Overall, most stakeholders believed they had medium levels of wild pigs on their property(ies), which had been slightly increasing over the past five years. Of the small number of respondents who stated wild pig population levels on their property(ies) had been decreasing, hunting, trapping and the neighboring properties management actions were believed to be the main causes. Contrastingly, of the large number of respondents who believed wild pig populations had been increasing on their property(ies), lack of hunting pressure, natural causes (e.g., increased food or water supply, dispersal from surrounding area), and ineffective state and/or federal management action was identified as the foremost reasons. Engagement in wild pig management was much lower than expected with less than half of survey respondents (~41%) stating that they had engaged in wild pig management within the last five years. Of those respondents that had participated in management, opportunistic shooting, hunting, and trapping were the three most utilized management techniques and deemed as neutral to somewhat effective at reducing wild pig populations.

When asked who should manage wild pigs in Alabama, stakeholders believed individual landowners, hunters, and state agencies were most responsible. Additionally, the individuals responsible for the illegal release of wild pigs, individual landowners, and insurance companies were most often selected as those responsible for paying for damages associated with wild pigs. The concerns of animal welfare groups (e.g., PETA), wild pig related businesses (e.g., guided pig hunts, removal companies, wild game meat processors), and the public were deemed to be of neutral to slight unimportance for authorities to take into consideration when addressing wild pig management questions. All groups found the current legal penalties for transporting or releasing live wild pigs in Alabama to be of sufficient severity. Lastly, respondents strongly believed that wild pigs are an issue because they are economically costly, threaten public health and safety, decrease wildlife habitat, reduce soil quality via rooting activities, and have negative impacts on all species of wildlife, predominantly white-tailed deer, turkey, and other game bird species (e.g., bobwhite quail, mourning dove).

Based on previous human dimensions literature (Messmer et al. 1997; Daigle et al. 2002; Lohr et al. 2014) that found that stakeholder perspectives significantly differ by the group they belong to, we had expected these groups (hunters, farmers, forest landowners) to be considerably dissimilar from one another, due to the variability in their interactions and experiences with wild pigs. However, farmers and forestland owners’ opinions on wild pig management, policy, and impact were quite similar, whereas hunters’ perspectives differed the most frequently from the other groups. This difference may be due to whether the respondent owned (or leased under their management) the property on which wild pig damage was observed. Hunters in our survey may not have been landowners and therefore only accessed lands during a portion of the year for hunting. As such, they likely have neither been exposed to wild pig damage that occurs throughout the year nor borne the full financial cost of this damage. Regarding the impact of wild pigs, all groups were very similar in believing that wild pigs have negative economic, human, and ecological health impacts. Of the 70 differences found between groups, only ~13% occurred between farmers and forestland owners. The remaining ~87% occurred between hunters and farmers (~46%) or hunters and forestland owners (~41%). Despite having a large number of statistically significant differences between groups, most were not sociologically significant, as had been found with stakeholder views on use of toxicants (TuckerWilliams et al. 2021). In no instance did one group’s perspective drastically conflict with another’s.

Interestingly, even though all groups indicated that they had seen a general increase in wild pig populations on their property(ies), less than half of respondents had actively participated in management efforts within the past five years. Stakeholders in Texas (Rollins 1993; Adams et al. 2005), Georgia (Mengak 2012), and Tennessee (Jerrolds et al. 2014) also perceived increases in wild pig populations on private property. In Georgia, the lack of hunting pressure and natural causes were the primary reasons for such increasing trends (Mengak 2012), which matches this study. Despite the perceived increase in wild pig populations on private land in Alabama, stakeholder participation in wild pig management was much lower compared to Texas and Illinois with 84% and 65% management participation, respectively (Adams et al. 2005; Harper et al. 2014). Of those that had participated in wild pig management, opportunistic shooting, hunting, and trapping were used most often, which is consistent with previous research (Adams et al. 2005; Higginbotham et al. 2006; Mengak 2012; Anderson et al. 2016).

As the primary method of management, hunting and opportunistic shooting have been shown to be ineffective at reducing wild pig populations (Jerrolds et al. 2014; Summers et al. 2017). While whole sounder removal via trapping is the most effective when executed properly (Sparklin et al. 2009; Smith et al. 2014), it can be initially daunting and costly, as well as extremely labor and time intensive, which could be why less than 60% of respondents who participated in wild pig management used it as a technique within the past five years. Additionally, simply because respondents are participating in trapping on their property does not signify trapping efforts are being done properly to achieve the greatest level of success. Collectively, however, the groups believed hunting and shooting over bait to be equally effective as trapping. Current wild pig management options were considered somewhat ineffective to somewhat effective by survey respondents, which are consistent with findings from Georgia (Mengak 2012), where respondents perceived that most management techniques were ineffective at reducing wild pig populations.

All groups agreed that individual landowners should be responsible for managing wild pigs. While landowners agreed they should be responsible for management on their land, no specifics were provided on what that management actually entailed. Our interpretation is that landowners feel they are responsible for what happens on their land, and therefore, are responsible for determining the management actions, whether that be by personally controlling wild pig populations or outsourcing and allowing access for management purposes. Local and state government action and agency removal efforts were thought to be the least responsible for decreasing wild pig populations in the state by all groups. Such perspectives most likely have to do with the fact that these organizations are only able to operate on state and federally owned and managed lands, unless granted access by private landowners. Therefore, private landowners are unaware of current control efforts and are much less likely to experience a benefit from such efforts on their property, unless it borders public lands. A similar study in Georgia found supporting evidence that approximately 50% of respondents sought outside assistance to address wild pig damage on their property, of which roughly 83% enlisted the help of private wild pig control and removal companies. Georgia Wildlife Resource Division and the USDA Wildlife Service were asked to assist by only approximately 20% and 5% of survey respondents, respectively (Mengak 2012).

Most respondents believed the individuals responsible for the introduction of wild pigs on their property(ies) should be responsible for compensation of associated damages. Because it is extremely difficult to prove who is responsible for releasing wild pigs without catching the perpetrators in the act, it is impractical to consider this a viable solution. Additionally, of the legal penalties associated with being found guilty of releasing wild pigs, all groups found the mandatory fine and jail time to be sufficient in severity. Forestland owners were generally split between believing the Class B misdemeanor was either too weak or a sufficient penalty. Interestingly, all groups were more supportive of individual landowners and insurance companies being responsible for paying for wild pig related damages on their property than the state or federal government. Such views may stem from stakeholders believing that they are responsible for their land and land management and a general lack of trust in the government as seen in other research (Raedeke et al. 2001; Gray et al. 2012; Caplenor et al. 2017).

Stakeholders only viewed animal welfare group concerns about wild pig management as unimportant to agency and policy decisions. As to be expected, hunters, farmers, forestland owners’ concerns were thought to be somewhat to very important. All groups thought that scientists, biologists, wildlife and land manager concerns to be somewhat important, indicating an overall support for science-based management and policy decisions. Concerns of the public and wild pig related businesses were of neutral importance to authorities by all groups. Because wild pigs neither impact individuals in urban areas in the same manner as rural areas, nor do urban residents actively participate in management of wild pigs on their property, their concerns were considered less important compared to groups who are directly affected by the species. Additionally, because a relatively small group of individuals are profiting off wild pig related businesses (e.g., guided hunts, Rollins 1993; Tolleson et al. 1995; Adams et al. 2005), their concerns surrounding wild pig management are seen as less important by all groups than most individuals who are being negatively impacted by wild pigs.

By asking stakeholders their perspectives on the economic, human health, and ecological impact wild pigs have, we can gauge the level of knowledge respondents have about the negative impacts associated with wild pigs, and better understand what specifically about wild pigs may motivate people to participate in management activities. Remarkably, in no instance did any group believe wild pigs had a positive impact. All groups believed wild pigs were costly to individuals and the state, which supports the findings of previous research (Adams et al. 2005; Higginbotham et al. 2006; Mengak 2012; Jerrolds et al. 2014; Anderson et al. 2016; Poudyal et al. 2017). This awareness comes as no surprise as wild pigs are well known for being costly agricultural pests, destroying crops, rooting up fields and subsequently breaking field equipment, etc. (Pimentel et al. 2005; Higginbotham et al. 2006; Anderson et al. 2016; Holderieath 2016). Similar to previous studies in Texas (Adams et al. 2005), Georgia (Mengak 2012; Harper et al. 2016), and Illinois (Harper et al. 2014; Harper et al. 2016), respondents believed wild pigs were vectors of disease to livestock, and a threat to public health (e.g., disease, water contamination) and safety (e.g., vehicle collisions). Interestingly though, hunters least believed that wild pigs as being vectors of disease to humans (e.g., swine brucellosis) compared to the other groups. This lack of knowledge is quite concerning considering that hunters are most likely to become infected due to their increased exposure to blood and other bodily fluids during the field dressing and butchering process (Harder and Basta 2007; Giurgiutiu et al. 2009; Jack et al. 2012). The lack of concern surrounding wild pigs transmitting disease to humans may be due to a lack of education and awareness within the hunting community.

All groups believed wild pigs were negatively impacting ecological health, which supports previous research indicating that stakeholders considered wild pigs to be environmentally harmful (Adams et al. 2005; Mengak 2012; Harper et al. 2014; Harper et al. 2016; Caplenor et al. 2017). Such a unified opinion is encouraging from a management and policy perspective as it indicates stakeholder beliefs are congruent with scientific evidence stating that wild pigs have negative impacts on water (Barrios-Garcia and Ballari 2012; Bolds et al. 2022) and soil quality and processes (Siemann et al. 2009; Barrios-Garcia and Ballari 2012), tree health (Hopkins 1947; Lipscomb 1989; Eckhardt et al. 2016), wildlife, and habitat (Engeman et al.^2003^; Jolley et al. 2010; Barrios-Garcia and Ballari 2012; Cole et al. 2012; Strickland et al. 2020; McDonough et al. 2022).

Lastly, of the ten species and categories of wildlife survey respondents were presented with, respondents believed that wild pigs had a negative impact on all of them. Predators and carnivores (e.g., coyote [*Canis latrans*], bobcat [*Lynx rufus*], and fox [*Urocyon cinereiargenteus*, and *Vulpes vulpes*]) were believed to be the least impacted, but still viewed as experiencing neutral to slight negative impacts associated with wild pigs. Interestingly, wild pigs were believed to have slight negative impacts on the non-primary game species and categories of wildlife (e.g., amphibians, endangered species, reptiles, small mammals) by all groups. The level of perceived negative impact on non-game species by respondents was much greater than that found by Mengak (2012). Because these stakeholder groups were likely more attuned to populations of preferred game species (e.g., white-tailed deer, turkey, bobwhite quail) on their property, their perspectives of wild pig impact on non-game species were encouraging.

Wild turkey, white-tailed deer, and other game birds (e.g., bobwhite quail and mourning dove) were believed to be the most severely impacted by wild pigs by all groups. Similar studies also identified these species as being negatively impacted by wild pigs (Rollins 1993; Tolleson et al. 1995; Adams et al. 2005; Mengak 2012; Harper et al. 2014). Again, this finding is unsurprising considering these are highly publicized, preferred game species of many stakeholders and are actively managed for on private and public lands. Therefore, stakeholders are more likely to be sensitive to any perceived negative associations between those species and wild pigs. The lack of perceived positive wildlife impacts associated with wild pigs is another advantage for managers and policy makers. Because game species such as the white-tailed deer, wild turkey, and bobwhite quail are such an economic powerhouses and culturally significant species in the Southeast (Burger et al. 1999; Grado et al. 2007; Munn et al. 2010), the perspective that wild pigs are negatively impacting those species could have major implications from a policy standpoint.

Limitations did occur during the project aside from the low response rates and lack of non-response bias testing. Within the first week of disseminating the survey, the Auburn University main servers experienced a fire, causing the servers to shut down and all associated Auburn networks to go offline for approximately 1 day, including Qualtrics. During this time survey respondents were unable to access the survey. Once Qualtrics was back online, an email was sent out to all potential survey respondents explaining the technical difficulty, encouraging potential respondents to try again and apologizing for the inconvenience. Unfortunately, testing for non-response bias was not possible based on the invitation and data collection requirements of both the software and human subjects’ requirements. Specifically, IP addresses of respondents were not collected to protect respondent anonymity in accordance with the Auburn University IRB. Therefore, we were unable to use that information for non-response bias testing. Additionally, because ALFA and AFOA did not want to release the contact information of their members, each organization sent the survey link in an email directly to their members. Again, because we lacked access to member email addresses in addition to IP addresses, there was no way to identify specifically who participated in the survey and who did not. Therefore, non-response bias testing was not possible. Due to the low response rate and inability to test for non-response bias, we are unable to claim these findings to be representative of each stakeholder group. However, these findings do help us to begin to develop a baseline understanding to assist future research into the topic.

## Management Implications

The findings of this research provide a more detailed comprehension of our current understand of stakeholders’ perspectives on wild pig policy, population trends, management occurring on privately owned land, and stakeholder beliefs on the economic, human health, and ecological impact of wild pigs. From a policy standpoint, the overall agreement amongst and between groups on the negative economic, human health, and ecological impacts associated with wild pigs indicates that stakeholders and science are non-conflicting, and therefore, of minimal hindrance to management. However, varying levels of management engagement, effort, control efficacy, and a haphazard patchwork of concentrated removal efforts between privately and publicly owned or managed lands does not facilitate sustainable and effective widespread wild pig management (Epanchin-Niell et al. 2010).

For impactful and sustainable management to happen, agencies, NGOs, academia, and private landowners need to collaborate and work towards improving management by effectively removing wild pigs systematically and monitoring for reinvasions on private lands (Epanchin-Niell et al. 2010; Glen et al. 2017; Pepin et al. 2017). Regional wild pig control could be obtainable by engaging landowners in management cooperatives and sharing in the cost and labor associated in removal efforts. Wild pig management cooperatives could be more appealing to landowners if they were provided access to rentable equipment or equipment at reduced costs and provided with technical assistance on how to effectively remove wild pigs over time and monitor for reinvasions. Finally, incentivizing continued absence or extremely low numbers of wild pigs on private property could help to increase the longevity of management efforts. One thing is for certain, without management collaboration between public land managers and private landowners at a landscape level, wild pigs will continue to be economic costly, ecological disastrous and threaten public health and safety into the foreseeable future on a global scale.

## Appendix 1. Survey Questions Pertaining to Stakeholders’ Perspectives on Wild Pig Management in Alabama. Answer Options are Presented within Parentheses with Associated Coding

1. In your opinion, the wild pig population in general across all of your properties is (fill in the blank). (1= low [I rarely see wild pigs on my property], 2 = medium [I sometimes see wild pigs on my property], 3 = high [I see wild pigs on my property frequently], treated as missing data = unsure)
2. In general over the past 5 years, the size of the wild pig population across all of your land has in your opinion… (1 = largely decreased, 2 = slightly decreased, 3 = stayed the same, 4 = slightly increased, 5 = largely increased)
3. Why do you think wild pig populations across all of your properties has generally decreased? (select all that apply) “hunting has reduced the population, trapping, agency removal efforts, state/local government action, neighboring properties management practices (e.g., burning, trapping), state regulations prohibiting the transportation of wild pigs, natural causes (e.g., decreased food sources, decreased water availability, dispersal of wild pigs from your area into other areas), habitat alterations (e.g., decreased density of forest understory, decreased vegetation alongside water sources), other (please specify)”
4. Why do you think the wild pig population across all of your properties has increased? (select all that apply) “Released/escaped from domestic pig producers, released by hunters to increase wild pig hunting opportunities, released by government agencies, neighboring property’s management practices (e.g., lack of removal efforts, lack of burning to reduce understory vegetation), lack of hunting pressure, natural causes (e.g., increased food sources, increased water supply, dispersal of wild pigs from surrounding area into your area), habitat alteration (e.g., increased density of forest understory, increased vegetation alongside water sources), lack of effective action taken by the state and/or federal agencies, other (please specify)
5. Have you engaged in any wild pig management within the last 5 years? (1 = yes, 2 = no)
6. Within the last 5 years, how have you managed for wild pigs? (select all that apply) “Hunting wild pigs (recreational or subsistence), shooting wild pigs (opportunistically, to control for damage, to manage populations), leased hunting rights to increase hunting pressure, trapped wild pigs (e.g., corral trap, box trap), fencing to exclude pigs from certain areas, including electric fences, scare tactics (e.g.. motion activated scarecrows scents to deter wild pigs), habitat alterations (reducing habitat to make property less appealing to wild pigs, such as removing understory), used trained dogs to hunt or harass wild pigs, hire wild pig removal specialists (paying money for a business to remove the wild pigs from the property), fed wild pigs to reduce damage to profitable items (e.g., row crops), provided resources with the intent to increase wild pig populations on any of your properties.”
7. Of the following current legal wild pig management options, please indicate how effective at reducing wild pig populations you believe them to be. “Nuisance permit for hunting, night shooting, trapping and lethal removal (e.g., corral traps), opportunistic shooting (not actively seeking out wild pigs but shooting them if the opportunity presents itself), wild pig management cooperatives (groups of landowners come together and share in the cost and labor of removing wild pigs from their collective land), for hire removal service, hunting/shooting over bait, aerial shooting by helicopter, fencing (including electric), scare tactics (e.g., motion activated scarecrows, scents to deter wild pigs), habitat alteration (e.g., burning to remove understory” (1 = very ineffective, 2 = somewhat ineffective, 3 = neutral, 4 = somewhat effective, 5 = very effective)
8. Please select the circle that represents the extent to which you agree or disagree with each of the following statements. “I believe that wild pigs… are an issue because they cost me money, are economically costly to the state, threaten human health (e.g., disease, water contamination), threaten public safety (e.g., vehicle collisions), have a positive ecological impact, reduce quality of water sources (e.g., stream bank erosion, reduce water quality), improve soil quality by rooting, cause tree loss/damage, improve wildlife habitat, other (please specify)” (1 = strongly disagree, 2 = somewhat disagree, 3 = neutral, 4 = somewhat agree, 5 = strongly agree)
9. The following statements are about the potential for disease or death due to wild pigs. Select the response that represents the extent to which you agree or disagree with each statement. “ I believe wild pigs…. kill newborn livestock, transmit disease to domestic livestock, transmit disease to humans” (1 = strongly disagree, 2 = somewhat disagree, 3 = neural, 4 = somewhat agree, 5 = strongly agree)
10. Please select the option that represents the extent to which you believe the following wildlife species are positively or negatively affected by wild pigs. “amphibians (e.g., salamanders, frogs), endangered species, fish, game birds (e.g., mourning dove, bobwhite quail), predators/carnivores (e.g., coyote, fox, bobcat, raccoon), reptiles (e.g., snakes, lizards, turtles), small mammals (e.g., squirrel, rabbit), waterfowl (e.g., ducks, geese), white-tailed deer, wild turkey, other (please specify)” (1 = strong negative impact 2 = slight negative impact, 3 = neutral/no impact, 4 = slight positive impact, 5 = strong positive impact, treated as missing data = unsure of impact)
11. Please indicate how strongly you agree or disagree with the following statements… “I believe wild pig populations throughout Alabama should be managed by… the Extension Service, federal agencies (e.g., USDA, Fish and Wildlife Service, hunters, individual land owners, the private industry (e.g., for hire wild pig removal companies), state agencies (e.g., Alabama Department of Conservation and Natural Resources), the general public, (1 = strongly disagree, 2 = somewhat disagree, 3 = neutral, 4 = somewhat agree, 5 = strongly agree)
12. Who do you believe should be responsible for paying for damages caused by wild pigs? (select all that apply) “The state of Alabama, the federal government, individual land owners who receive the damage, insurance companies (e.g., crop insurance, property insurance), public in the form of taxes.”
13. Please state how important you believe it is for authorities (e.g., Alabama Department of Conservation and Natural Resources) to address the concerns of the following groups of people regarding wild pig management in Alabama. “Animal welfare groups (e.g., The Humane Society, PETA), biologists, wildlife/land managers, scientists, farmers/agriculture professionals, forestland owners, hunters, land owners, the general public, public land recreational uses (e.g., hikers, birders, horseback riders), wild pig related businesses (e.g., guided pig hunts, removal companies, wild game meat processors)” (1 = extremely unimportant, 2 = somewhat unimportant, 3 = neutral, 4 = somewhat important, 5 = extremely important)
14. Currently it is illegal to transport or release live wild pigs anywhere in Alabama due to their destructive nature and potential to transmit disease. If caught, an individual will be charged with a Class B misdemeanor, mandatory fine of $2500 per wild pig, and up to 180 days in jail. Please tell us how sufficient or insufficient you feel each of these components are for penalizing individuals caught transporting or releasing live wild pigs in Alabama. “Class B misdemeanor, mandatory fine of $2500 per wild pig, up to 180 days in jail” (1 = no, the penalty needs to be stronger, 2 = yes, this is a sufficient penalty, 3 = no, the penalty needs to be stronger)
